# UK Biobank’s cardiovascular magnetic resonance protocol

**DOI:** 10.1186/s12968-016-0227-4

**Published:** 2016-02-01

**Authors:** Steffen E. Petersen, Paul M. Matthews, Jane M. Francis, Matthew D. Robson, Filip Zemrak, Redha Boubertakh, Alistair A. Young, Sarah Hudson, Peter Weale, Steve Garratt, Rory Collins, Stefan Piechnik, Stefan Neubauer

**Affiliations:** 1 William Harvey Research Institute, NIHR Cardiovascular Biomedical Research Unit at Barts, Queen Mary University of London, Charterhouse Square, London, EC1M 6BQ UK; 2 Division of Brain Sciences, Department of Medicine, Imperial College, London, UK; 3 Division of Cardiovascular Medicine, Radcliffe Department of Medicine, University of Oxford, Oxford, UK; 4 Department of Anatomy and Medical Imaging, University of Auckland, Auckland, New Zealand; 5 UK Biobank, Spectrum Way, Adswood, Stockport, Cheshire SK3 0SA UK; 6 Siemens Healthcare, Frimley, Surrey GU16 8QD UK

## Abstract

**Background:**

UK Biobank’s ambitious aim is to perform cardiovascular magnetic resonance (CMR) in 100,000 people previously recruited into this prospective cohort study of half a million 40-69 year-olds.

**Methods/design:**

We describe the CMR protocol applied in UK Biobank’s pilot phase, which will be extended into the main phase with three centres using the same equipment and protocols. The CMR protocol includes white blood CMR (sagittal anatomy, coronary and transverse anatomy), cine CMR (long axis cines, short axis cines of the ventricles, coronal LVOT cine), strain CMR (tagging), flow CMR (aortic valve flow) and parametric CMR (native T1 map).

**Discussion:**

This report will serve as a reference to researchers intending to use the UK Biobank resource or to replicate the UK Biobank cardiovascular magnetic resonance protocol in different settings.

## Background

Understanding the determinants of diseases, such as myocardial infarction and stroke, is critical to advance medical knowledge that can lead to prolongation of life and improvement in quality of life. A combination of various risk factors often leads to disease with each risk factor having only moderate effects that also interact with each other in complex ways. Prospective cohort studies allow insights into risk factors before disease develops or into how disease management affects participants.

## Methods/Design

UK Biobank, a prospective cohort study of half a million 40-69 year-olds (mean age 56.5 years, 54.4 % female, 94.4 % White, 1.9 % Asian/Asian British, 1.6 % Black/Black British), started to recall participants for a comprehensive imaging visit. Baseline summary characteristics of the cohort can be viewed in the data showcase on UK Biobank’s Web site (www.ukbiobank.ac.uk).

The imaging visit includes a 35-min brain magnetic resonance imaging (MRI) scan at 3 Tesla, a dual energy X -ray absorptiometry (DEXA) scan (10–15 min), and carotid ultrasound (10–15 min) in addition to preparation (including consenting) and collection of non-imaging data and biological samples (e.g. partial repeat of the baseline assessment visit with supplementary cognitive function tests), and 20-min cardiovascular magnetic resonance (CMR) at 1.5 Tesla and 10-min abdominal MRI also at 1.5 Tesla (average time over 3 month period was 30 min for both parts combined at 1.5 T Tesla). This resulted in an average total visit time of 3 h 29 mins.

The rationale, challenges and approaches to perform 100,000 CMR scans in UK Biobank have been described elsewhere [1]. The pilot phase is almost completed with more than 7300 participants as of early January 2016 (see the current subject counter displayed on www.ukbiobank.ac.uk). A first release of these imaging (with associated phenotypic) data occurred in October 2015.

The purpose of this article is to describe the details of the relevant methodology for CMR acquisition during the first 20 months of the pilot phase, which started in May 2015. This article aims to serve researchers as a source of information for planning access applications (www.ukbiobank.ac.uk/register-apply/) to this shared resource and to serve as a reference for publications arising from the use of UK Biobank CMR data.

The CMR protocol was developed bearing in mind the set-up and requirements described previously [1]. In brief, each participant undergoes a 20-min CMR protocol without pharmacological stressor or contrast agent, as part of a 30- min combined CMR and abdominal MRI protocol.

### Cardiovascular magnetic resonance infrastructure

CMR imaging is being performed in Cheadle, United Kingdom, on a clinical wide bore 1.5 Tesla scanner (MAGNETOM Aera, Syngo Platform VD13A, Siemens Healthcare, Erlangen, Germany). The scanner is equipped with 48 receiver channels, a 45 mT/m and 200 T/m/s gradient system, an 18 channels anterior body surface coil used in combination with a 12 elements of an integrated 32 element spine coil and electrocardiogram (ECG) gating for cardiac synchronization.

In addition to the vendor’s advanced cardiac package, the Shortened Modified Look-Locker Inversion recovery technique (ShMOLLI, WIP780B) is implemented on the scanner in order to perform native (non-contrast) myocardial T1 mapping. The Cardiac Dot Engine (Siemens Healthcare, Erlangen, Germany) is used to facilitate quality for consistency of image acquisition throughout the study.

### CMR protocol

**Fig. 1:**
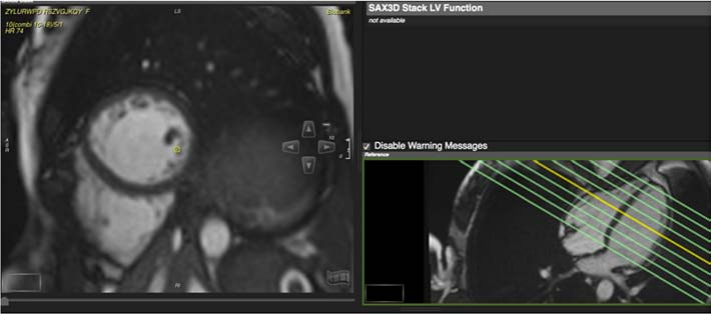
Planning of the short axis cine stack covering the entire left and right ventricles

**Fig. 2:**
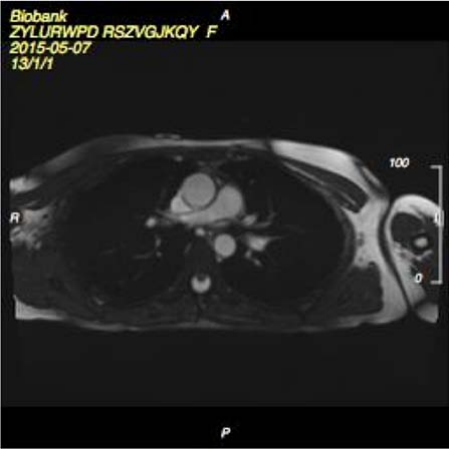
Transverse aortic cine at the level of the pulmonary trunk/right pulmonary artery

UK Biobank’s CMR acquisitions include piloting and sagittal, transverse and coronal partial coverage of the chest and abdomen. For cardiac function, three long axis cines (horizontal long axis – HLA, vertical long axis – VLA, and left ventricular outflow tract –LVOT cines both sagittal and coronal) and a complete short axis (SA) stack of balanced steady state free precession (bSSFP) cines, covering the left ventricle (LV) and right ventricle (RV) are acquired (Fig. 1). Aortic compliance can be derived from a transverse bSSFP cine at the level of the pulmonary trunk and right pulmonary artery (Fig. 2). Immediately before and after this bSSFP acquisition of the aorta, brachial blood pressure readings are being obtained using a manual sphygmomanometer used for calibrating peripheral waveforms and immediately afterwards a brachial pressure wave trace is digitally computed by the Vicorder (Skidmore Medical, Bristol, UK) with the cuff statically inflated to 70 mmHg using a volume displacement technique. The Vicorder software calculates values for central blood pressure by applying a previously described brachial-to-aortic transfer function [2]. Aortic distensibility represents the relative change in area of the aorta per unit pressure, taken here as the central pulse pressure and is calculated according to the formula:

$$ \mathrm{aortic}\ \mathrm{distensibility}=\left({\mathrm{A}}_{\max}\hbox{-} {\mathrm{A}}_{\min}\right)/{\mathrm{A}}_{\min }/\left({\mathrm{P}}_{\max}\hbox{-} {\mathrm{P}}_{\min}\right), $$

where A_max_ = maximal (systolic) area (mm^2^), A_min_ = minimal (diastolic) area (mm^2^), P_max_ = systolic blood pressure (mm Hg), and P_min_ = diastolic blood pressure (mm Hg) [3].

**Fig. 3:**
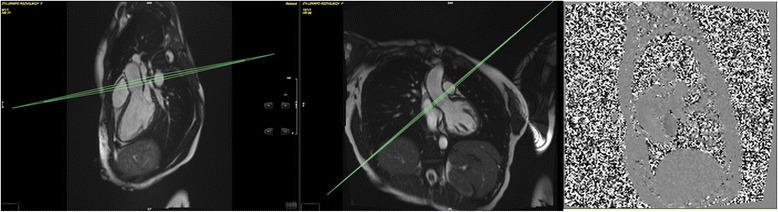
Aortic valve flow imaging view planned using the sagittal and coronal left ventricular outflow tract (LVOT) cines

A phase contrast sequence is planned on both sagittal and coronal LVOT cines to capture aortic flow and the number of valve cusps (Fig. 3). The plane is located at or immediately above the sino-tubular junction at end diastole as recommended [4]. The standard velocity encoding (VENC) is 2 m/s but is adjusted upwards based on presence/degree of turbulence seen on the LVOT cines and if time allows. Tagging (grid) is acquired in 3 short axis views (basal, midventricular and apical) carefully avoiding the LVOT in the basal slice. The midventricular slice position of the tagging matches the native T1 mapping short axis slice. An international expert advisory group helped to guide adjustments made to the originally planned protocol [1] to ensure that the protocol could be consistently conducted within 20 min, but these have not compromised the information available. The atrial short axis cine stack has been removed; atrial end-diastolic and end-systolic volumes and atrial ejection fraction can be measured with the long-axis cines. Three long-axis tagged cines have been removed to save time, but the radial and longitudinal strain parameters can be derived reliably using feature-tracking techniques from the untagged long-axis cines acquired. A phase contrast sequence (~1 min) was added to allow aortic flow and number of aortic cusps to be captured, adding scientific value, since aortic stenosis is the most common heart valve lesion, has a poor prognosis if severe and is increasingly common in ageing populations. Also native T1 mapping in one midventricular short axis was added to allow myocardial tissue characterisation without the use of contrast agents. The details of the CMR sequences are summarised in Table 1.

**Table 1:** Cardiovascular magnetic resonance protocol for UK Biobank

Description	Sagittal anatomy	Coronal and Transverse anatomy”	Long axis cines	Short axis cines	Aortic distensibility cine	Tagging	Coronal LVOT cine	Aortic valve flow	Native T1 map
Pulse sequence: Simplified terminology (scientific terminology)	White blood CMR (bSSFP)	White blood CMR (bSSFP)	Cine CMR (bSSFP)	Cine CMR (bSSFP)	Cine CMR (bSSFP)	Strain CMR (GRE)	Cine CMR (bSSFP)	Flow CMR (GRE)	Parametric CMR (ShMOLLI WIP780B)
Flip angle (°)	80	80	80	80	80	12	80	20	35
TR (ms)	2.6	2.6	2.7	2.6	2.8	8.2	2.7	4.6	2.6
TE (ms)	1.12	1.12	1.16	1.10	1.17	3.90	1.16	2.47	1.07
GRAPPA factor	2	2	2	2	2	0	2	2	2
Slice thickness (mm)	8.0	8.0	6.0	8.0	6.0	8.0	6.0	6.0	8.0
Slice gap (mm)	2.64	4	n.a.	2	n.a.	n.a.	n.a.	n.a.	n.a.
Typical Field of View (mm)	400 x400	400 x 400	380 x 274	380 x 252	380 x 294	350 x 241	380 x 384	340 x 340	360 x 236
Matrix	240 x 158	240 x 158	208 x 187	208 x 187	240 x 216	256 x 174	208 x 187	192 x 173	192 x 192
Voxel size	1.7 x 1.7 x 8.0	1.7 x 1.7 x 8.0	1.8 x 1.8 x 6.0	1.8 x 1.8 x 8.0	1.6 x 1.6 x 6.0	1.4 x 1.4 x 8.0	1.8 x 1.8 x 6.0	1.8 x 1.8 x 6.0	0.9 x 0.9 x 8.0 (Interpolation = On, factor 2)
Acquired temporal resolution (ms)	n.a.	n.a.	32.64	31.56	28.00	41.05	32.64	37.12	368.28
Calculated cardiac phases	1	1	50	50	50	1	50	30	1
ECG triggering/gating	PT	n.a.	RG	RG	RG	PT	RG	RG	PT
Other parameters			Inline Evaluation Ventricular Function	Inline Evaluation Ventricular Function		Grid spacing 6 mm, shared phases			T1 map determined on-line.
Image Filter	Off	Off	Off	Off	Off	Off	Off	Off	Off
Distortion Corr	On (2D)	On (2D)	On (2D)	On (2D)	On (2D)	On (2D)	On (2D)	On (2D)	Off
Raw Filter	Off	Off	Off	Off	Off	Off	Off	Off	On: Weak, slope 25
Elliptical filter	Off	Off	Off	Off	Off	Off	Off	Off	Off
No of breath-holds (expiration)	1	1	1 slice per breath-hold	1 slice per breath-hold	1	1 slice per breath-hold	1	1	1
Orientation	Sagittal (x11) PE direction = AP	Coronal (x10), Transverse (x10) PE direction = RL & AP	HLA, VLA, LVOT (sagittal) views PE direction = varies	Coverage based to apex in SA views (approximately x10) PE direction = AP	Transverse at level of pulmonary trunk/right pulmonary artery PE direction = AP	SA views (b, m, a) PE direction = AP	LVOT (coronal) view PE direction = RL	Aortic valve plane planned on both LVOT cines PE direction = AP	SA (m) PE direction = AP
Image example									

Abbreviations: *bSSFP* balanced steady state free precession, *PT* Prospective Triggering, *RG* Retrospective Gating, *b* basal, *m* midventricular, *a* apical, *HLA* Horizontal long axis, *VLA* Vertical Long Axis, *LVOT* Left Ventricular Outflow Tract, *SA* Short Axis

### CMR image analysis

Since the time of first data release, researchers are able to access the DICOM CMR image files, but only a limited range of measures derived from images is currently available. The automated inline ventricular function option is enabled on the scanner, which provides automatic assessment of LV contours and volumes. Given that UK Biobank provides LV end-diastolic volume, LV end-systolic volume, LV stroke volume, cardiac output and cardiac index to researchers without checking the endocardial and epicardial contours for quality it may be recommended that these are quality checked as the data application requires. A British Heart Foundation (BHF) project grant (PG/14/89/31194, PI Petersen, 6/2015 to 5/2018) currently funds the manual analysis to create a CMR reference standard for the UK Biobank imaging resource in 5000 CMR scans. Table 2 provides an updated list of derived CMR parameters that will be returned to the UK Biobank resource upon completion of the analysis. A UK Biobank CMR Image Analysis Consortium has been formed and has been planning issues around standardization and automating CMR image analysis. The work of the consortium expects to reduce the overall costs associated with cardiovascular image analysis for large-scale cohort studies through knowledge sharing, coordination of efforts and capacity building. An important aspect of the user data access agreement with UK Biobank is that measures derived in the course of individual research projects that are of general value to the research community will be returned to UK Biobank to be incorporated into the database (with a full description of the methods used) for others in the research community to access after an appropriate embargo period. Through this mechanism, for example, the CMR Image Analysis Consortium will return to the UK Biobank resource the manually segmented cardiac data and measures of the quality of the inline ventricular function contours expressed as calibration corrections for the automated tools relative to the manual analyses.

**Table 2:** The minimum dataset from the CMR images analyzed manually in the first 5,000 CMR scans will be returned to the UK Biobank resources and will likely include

Cardiovascular structure	Quantifiable parameters
Left ventricle	Myocardial mass (g), ejection fraction (%), end-diastolic volume (ml), end-systolic volume (ml), stroke volume (ml) and the corresponding values indexed to body surface area, height or weight. Time to peak contraction and filling rates (both in s) and peak contraction and filling rates (both in ml/s) can be derived from the cine images accepting the limitation of limited temporal resolution for this purpose. American Heart Association (AHA) myocardial segments: end-diastolic thickness (mm), end-systolic thickness (mm), thickening (mm), thickening (%). Strain (%) and strain-rates (%/s) in three directions (radial [ERR], circumferential [ECC] and longitudinal [ELL]) and in systole and diastole and corresponding changes in angle caused by shear (ERC and ECL). Torsion-rates (degrees/s). Midwall midventricular native T1 (ms).
Right ventricle	Myocardial mass (g), ejection fraction (%), end-diastolic volume (ml), end-systolic volume (ml), stroke volume (ml) and the corresponding values indexed to body surface area, height or weight
Left atrium	End-diastolic area (cm2), end-systolic area (cm2) and left atrial diameters as typically measured by 2D- echocardiography approaches. Using the area length formula atrial volumes (ml), atrial stroke volume (ml) and atrial ejection fraction (%) can be derived.
Right atrium	End-diastolic area (cm2), end-systolic area (cm2) and right atrial diameters as typically measured by 2D- echocardiography approaches. Using the area length formula atrial volumes (ml), atrial stroke volume (ml) and atrial ejection fraction (%) can be derived.
Aorta	Distensibility (1/mmHg) in ascending aorta and proximal descending aorta; diastolic aortic dimensions (cm2): aortic root (annulus, sinus of valsalva, sinutubular junction) ascending aorta and proximal descending aorta. Diameter of abdominal aorta as visualized.
Aortic valve	Peak velocity (cm/s), peak gradient (mmHg), forward volume (ml), regurgitant volume (ml), regurgitant fraction (%). Number of cusps of aortic valve (bicuspid, tricuspid, quadricuspid).

It is likely that different groups requesting access to UK Biobank will analyse images differently. Given this background we will not provide a detailed description of CMR image analysis approaches in this manuscript.

## Discussion

We describe the CMR protocol applied in UK Biobank’s pilot phase which will also be applied when UK Biobank extends this into the main phase with three centres using the same equipment and protocols. This manuscript will serve as a reference to researchers intending to use the UK Biobank resource for cardiac analyses or those who wish to replicate the UK Biobank CMR protocol in other settings.

## Abbreviations

bSSFP: Balanced steady state free precession
BHF: British Heart Foundation
CMR: Cardiovascular magnetic resonance
DEXA: Dual energy X -ray absorptiometry
ECG: Electrocardiogram
HLA: Horizontal long axis
LV: Left ventricle
LVOT: Left ventricular outflow tract
RV: Right ventricle
SA: Short axis
VENC: Velocity encoding
VLA: Vertical long axis
